# A rare diagnosis of abdominal pain presentation in the emergency department: Idiopathic omental bleeding: A case report

**DOI:** 10.1097/MD.0000000000009463

**Published:** 2017-12-22

**Authors:** Yen-Hung Wu, Kuan-Ting Liu, Chun-Kai Wen

**Affiliations:** a Department of Emergency Medicine, Kaohsiung Medical University Hospital; b School of Medicine, College of Medicine, Kaohsiung Medical University, Kaohsiung, Taiwan.

**Keywords:** abdominal pain, acute abdomen, idiopathic omental bleeding

## Abstract

**Rationale:**

Idiopathic omental bleeding is a rare cause of acute abdomen, with only a few reported cases. It usually presents with abdominal pain and may be life-threatening. As it rarely occurs, it may not be considered initially during patient presentation.

**Patient concerns:**

A 35-year-old male came to our emergency department with abdominal pain present for around 5 to 6 hours. The patient complained of left upper quadrant abdominal pain after eating breakfast. The only associated symptom was 3 episodes of vomiting up food. Physical examination revealed mild left upper quadrant abdominal tenderness without muscle guarding or rebounding pain. Blood examination showed leukocytosis with neutrophil predominance and C reactive protein elevation. The pain was persistent and relief was not obtained by medication.

**Diagnoses:**

Computed tomography showed a large lobular-contour homogenous slightly hyperdense lesion without enhancement along the greater curvature of the stomach in the lesser sac. A surgeon was consulted and laparotomy was suggested. Hematoma was found at Morrison pouch, subsplenic fossa, and lesser sac under operation.

**Intervention:**

Laparotomy and ligation for hemostasis.

**Outcomes:**

The patient was discharged with stable condition after 7 days of hospitalization.

**Lessons:**

This diagnosis should be considered in patients presenting with epigastric pain and vomiting after eating while in the emergency department because this disease might be life-threatening. This case highlights 2 important learning points. First, idiopathic omental bleeding could occur after eating in patients without underlying disease or trauma history, and this disease should be taken into consideration when acute abdomen occurs. Second, emergent laparotomy is indicated if the cause of acute abdomen is not clear.

## 1 Introduction

Idiopathic omental bleeding is a rare cause of acute abdomen, with only a few reported cases.^[1–4]^ It usually presents with abdominal pain and may be life-threatening. As it rarely occurs, it may not be considered initially during patient presentation. Here, we report a case visiting the emergency department because of abdominal pain after eating breakfast, and idiopathic omental bleeding was diagnosed. After, we contacted the regulations of institutional review board of the Kaohsiung Medical University Hospital, there was no need for ethical approval for this case report article. Informed consent was obtained from the patient.

## 2 Case presentation

**Figure 1:**
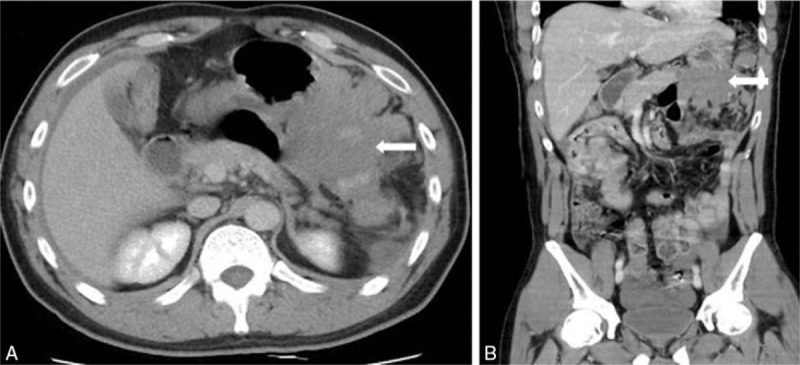
(A) Transverse view and (B) axial view of abdominal computed tomography with enhancement showed a large lobular-contour homogenous slightly hyperdense lesion without enhancement along the greater curvature of stomach in the lesser sac.

A 35-year-old male without any systemic disease and denying any medication use came to our emergency department with abdominal pain present for around 5 to 6 hours. When he arrived at emergency department, his consciousness was clear and vital signs showed body temperature 36.9° Celsius, blood pressure 112/73 mm Hg, with heart beat 102 per minute. The patient complained of left upper quadrant abdominal pain after eating breakfast. The only associated symptom was 3 episodes of vomiting up food. Physical examination revealed mild left upper quadrant abdominal tenderness without muscle guarding or rebounding pain. Blood examination showed leukocytosis (white blood cell: 15,980/μL with neutrophil predominance) and C reactive protein 5.59 mg/L, with normal renal and liver function. The pain was persistent and relief was not obtained by medication, then computed tomography was done, which showed a large lobular-contour homogenous slightly hyperdense lesion without enhancement along the greater curvature of the stomach in the lesser sac (Fig. 1A and B). A surgeon was consulted and laparotomy was suggested. Hematoma was found at Morrison pouch, subsplenic fossa, and lesser sac under operation, and 1000 mL bloody ascites were removed by suction, no obvious tumor lesion was found, bleeding from lesser omentum near spleen hilar was impressed, so ligation for hemostasis was performed. After the operation, the patient was discharged with stable condition after 7 days of hospitalization.

## 3 Discussion

Omental bleeding results from rupture of omental vessels, and it could be caused by tumor,^[1]^ trauma, aneurysm,^[2–3]^ coagulopathy,^[4]^ or vasculitis.^[5]^ Idiopathic omental bleeding is a rare cause. Omental bleeding often presents as abdominal pain, especially over the epigastric area, and is accompanied with nausea, vomiting, diarrhea, or even unstable hemodynamics. Some patients suffer just after a meal; the reason could be due to increased visceral blood flow after ingesting food.^[6]^ A larger meal increases the visceral blood flow and results in vessel rupture. The management of omental bleeding includes laparotomy or laparoscopy with omentectomy or simple vessel ligation, and transcatheter arterial embolization.^[7–9]^ In recent years, more minimally invasive surgery has been used such as transcatheter arterial embolization or laparoscopy.

In this case, the patient presented with symptoms of epigastric pain and vomiting after eating breakfast. No comorbidity was known before, and no trauma history or coagulopathy was noted. Emergent laparotomy was done instead of laparoscopy or transcatheter arterial embolization under the impression of massive intraperitoneal hemorrhage, with cause to be determined. Pathological examination of specimen revealed hemorrhage and showed no evidence of vasculitis, thrombosis, or malignancy. Idiopathic omental bleeding was thus diagnosed. In a review of the literature, idiopathic omental bleeding occurs more frequently in Japan than in other countries, and the occurrence of idiopathic omental bleeding ranges from young to old patients.^[10]^ Men are likely to suffer more than women from this condition.^[7–8,11–15]^ The reasons why there are more Japanese patients and why men are more likely than women to experience this condition are still unknown.

In conclusion, idiopathic omentum hemorrhage is a rare cause of acute abdomen which sometimes occurs after eating, while laparotomy with ligation and transcatheter artery embolization can be used to rule out malignancy and aneurysm. This diagnosis should be considered in patients presenting with epigastric pain and vomiting after eating while in the emergency department because this disease might be life-threatening. This case highlights 2 important learning points. First, idiopathic omental bleeding could occur after eating in patients without underlying disease or trauma history, and this disease should be taken into consideration when acute abdomen occurs. Second, emergent laparotomy is indicated if the cause of acute abdomen is not clear.
